# Clinical factor‐based risk stratification for precision therapy in locally advanced squamous cell carcinoma of the uterine cervix

**DOI:** 10.1002/cam4.6746

**Published:** 2024-01-08

**Authors:** Feng‐Yuan Liu, Wei‐Chun Chen, Yu‐Bin Pan, Chun‐Chieh Wang, Yun‐Hsin Tang, Hung‐Hsueh Chou, Angel Chao, Lan‐Yan Yang, Chyong‐Huey Lai

**Affiliations:** ^1^ Department of Nuclear Medicine and Molecular Imaging Center Chang Gung Memorial Hospital at Linkou and Chang Gung University College of Medicine Taoyuan Taiwan; ^2^ School of Medicine, National Tsing Hua University Hsinchu Taiwan; ^3^ Gynecologic Cancer Research Center Chang Gung Memorial Hospital at Linkou and Chang Gung University College of Medicine Taoyuan Taiwan; ^4^ Department of Obstetrics and Gynecology New Taipei City Municipal Tucheng Hospital New Taipei City Taiwan; ^5^ Department of Obstetrics and Gynecology Chang Gung Memorial Hospital at Linkou and Chang Gung University College of Medicine Taoyuan Taiwan; ^6^ Clinical Trial Center, Chang Gung Memorial Hospital Taoyuan Taiwan; ^7^ Department of Radiation Oncology Chang Gung Memorial Hospital at Linkou and Chang Gung University College of Medicine Taoyuan Taiwan

**Keywords:** ^18^F‐FDG PET/CT, concurrent chemoradiotherapy, locally advanced cervical cancer, risk stratification, squamous cell carcinoma

## Abstract

**Background:**

Concurrent chemoradiotherapy (CCRT) is the standard of care for locally advanced cervical cancer. In this study, we analyzed the pretreatment clinical and ^18^F‐fluorodeoxyglucose positron emission tomography/computed tomography (PET/CT) characteristics of patients with locally advanced cervical squamous cell carcinoma (SCC) to develop a scoring prototype for risk stratification.

**Methods:**

Two cohorts were constructed in this study. Cohort 1 comprised patients with cervical SCC with 2009 FIGO stage III‐IVA or stage I–II with positive pelvic or para‐aortic lymph node (PALN) on PET/CT from AGOG09‐001 trial. Cohort 2 comprised patients with similar characteristics who had received adequate therapy in our hospital between 2016 and 2021. Pretreatment patient characteristics and PET/CT parameters including maximum standardized uptake value (SUV_max_) and metabolic tumor volume (MTV) of primary tumor and nodal SUV_max_ were assessed for cancer‐specific survival (CSS) using multivariate Cox regression.

**Results:**

Analysis of combined data from cohorts 1 (*n* = 55) and 2 (*n* = 128) indicated age ≥ 66 years, primary tumor MTV ≥87 mL, and positive PALN on PET/CT to be independently significant adverse predictors for CSS (*p* < 0.001, *p =* 0.014, and *p =* 0.026, respectively) with a median follow‐up duration of 51 months. Assigning a score of 1 to each adverse predictor, patients with cumulative risk scores of 0, 1, 2, and 3 were discovered to have a 5‐year CSS of 86.9%, 71.0%, 32.2%, and 0%, respectively (*p* < 0.001).

**Conclusion:**

Age, primary tumor MTV, and positive PALN on PET/CT may serve as independent predictors of poor survival in patients with locally advanced cervical SCC. Our findings indicate that patients without any adverse factors can receive standard CCRT, whereas those with at least one adverse factor can consider novel combination therapies or clinical trials.

## Graphical abstract

This article describes a scoring prototype for risk stratification and precision therapy for locally advanced cervical cancer. Different therapies should be considered for patients with different cumulative risk scores.

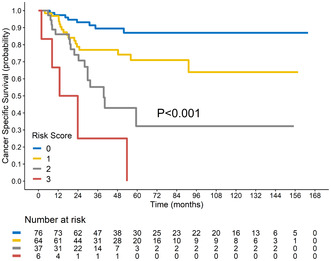

## 1 INTRODUCTION

Cervical cancer is the fourth most common cancer in women worldwide.^1^ Since the publication of benchmark clinical trials and the issuance of the National Cancer Institute's clinical alert in 1999, concurrent chemoradiotherapy (CCRT) has become the standard of care for patients with locally advanced cervical cancer (LACC) without distant metastasis.^2^ A phase III randomized clinical trial (RCT) conducted by Dueñas‐González et al. indicated that survival could be improved in the treatment of International Federation of Gynecology and Obstetrics (FIGO) stage IIB‐IVA cervical cancer by supplementing CCRT with weekly gemcitabine and two cycles of post‐CCRT adjuvant chemotherapy. However, this regimen has not been accepted as a standard of care.^3^ Subsequent efforts toward improving clinical outcomes in patients with LACC have also been unsuccessful.^4^, ^5^, ^6^ In the Asian Gynecologic Oncology Group (AGOG) 09‐001 trial, gemcitabine was added to cisplatin during CCRT in comparison to single‐agent cisplatin in patients with 2009 FIGO stage III–IVA or any stage with pelvic lymph node (PLN) or para‐aortic lymph node (PALN) metastasis. However, this regimen failed to improve the 3‐year progression‐free survival (PFS) (71.0% vs. 65.1%, *p* = 0.71) and overall survival (OS) (85.9% vs. 74.1%, *p* = 0.89).^4^ The phase III OUTBACK trial added 4 courses of paclitaxel and carboplatin after CCRT in a similar group of patients, but it also failed to improve PFS and OS.^5^

In 2014, the US Food and Drug Administration (FDA) approved the use of bevacizumab, an angiogenesis inhibitor, for the treatment of recurrent/persistent and advanced cervical cancer. The Gynecologic Oncology Group 240 RCT revealed that the addition of bevacizumab improved OS in persistent/recurrent or metastatic cervical cancer with a hazard ratio (HR) of 0.77.^7^ Although the role of bevacizumab in LACC remains uncertain, its administration at a dose of 10 mg/kg every 2 weeks for 3 cycles in addition to weekly administration of cisplatin at a dose of 40 mg/m^2^ during CCRT led to a 3‐year PFS of 81.3% and OS of 68.7% in the phase II Radiation Therapy Oncology Group 0417 trial.^8^ Another angiogenesis inhibitor, Endostar, which works through the inhibition of VEGFR2, has been demonstrated to improve the efficacy of CCRT in LACC with VEGFR2 expression.^9^ Pembrolizumab, in combination with chemotherapy, has been approved by the US FDA for the treatment of patients with persistent/recurrent or metastatic cervical cancer with PD‐L1 expression due to the results of phase III KEYNOTE‐826 trial.^10^ This RCT demonstrated that the addition of pembrolizumab improved OS with a HR of 0.64. Thus, the addition of immune checkpoint inhibitors to the therapeutic regimen of patients with LACC is likely to be beneficial. However, the phase III CALLA trial, which used durvalumab in combination with and following CCRT, failed to improve survival.^6^ Another phase III RCT (ENGOT‐cx11/KEYNOTE‐A18) for evaluating the benefits of using pembrolizumab with CCRT in the treatment of patients with LACC is currently ongoing.^11^ With the advancement of novel therapies, reliable strategies are required for risk stratification in patients with LACC. Although the cancer prognosis may be favorable and standard CCRT may be adequate for some patients, others may have an unfavorable prognosis; these patients should consider novel combination therapies.

Molecular imaging utilizing ^18^F‐fluorodeoxyglucose (^18^F‐FDG) and positron emission tomography (PET), which is usually integrated with computed tomography (CT), is widely performed for examining patients with cervical cancer.^12^ In addition to facilitating tumor staging, pretreatment ^18^F‐FDG PET/CT offers functional insights into tumors; for example, it provides information regarding the maximum standardized uptake value (SUV_max_) and volumetric measures such as metabolic tumor volume (MTV). The prognostic value of SUV_max_ in cervical cancer has been previously assessed in a meta‐analysis.^13^

In the present study, we developed a scoring prototype for risk stratification in patients with locally advanced primary cervical squamous cell carcinoma (SCC). This was achieved by analyzing the clinical and PET characteristics of the patients.

## 2 METHODS

### 2.1 Study cohorts

In the prospective, randomized AGOG09‐001 trial, patients with locally advanced primary cervical SCC were enrolled between 2009 and 2012 to participate in an investigation of whether the addition of gemcitabine to CCRT can improve survival.^4^ A parallel imaging study involving 55 patients demonstrated that ^18^F‐FDG PET/CT performed before and during treatment can help predict treatment failure and OS.^14^ However, during‐treatment parameters are unavailable for clinical cases in general. Only pretreatment predictors are available for risk stratification for patients with LACC. For the current study, we updated the survival data in the AGOG09‐001 study (cohort 1) and added patients with similar clinical status who underwent pretreatment PET/CT and received adequate therapy in our institution between 2016 and 2021 (cohort 2) for evaluation.

Both cohorts comprised of cervical SCC patients with either 2009 FIGO stage III‐IVA or stage I‐II with positive PLN or PALN on PET/CT.^15^ In cohort 1, the following patients were excluded: patients aged <35 or > 70 years; those with inadequate bone marrow or impaired pulmonary, liver, or renal function; those with an Eastern Collaborative Oncology Group performance status of >1; and those with a history of chemotherapy or pelvic radiotherapy. In cohort 2, patients who did not receive adequate therapy were excluded since inadequate therapy might exert confounding effects on survival. This study was approved by the Chang Gung Medical Foundation Institutional Review Board (202200908B0). The requirement for written informed consent was waived because of the retrospective nature of this study.

### 2.2 ^18^F‐FDG PET/CT imaging

Two PET/CT machines (Discovery ST16, GE Healthcare; Biograph mCT, Siemens Healthcare) were used. The patients fasted for 6 h before undergoing examination. Imaging was performed 50 min after an intravenous injection of ^18^F‐FDG (370 MBq ± 10%). A nonenhanced CT scan from the head to the thighs was performed; this was followed by PET imaging. All images were acquired with the patient in the supine position. On the basis of the CT data, the PET data were corrected for attenuation by using an ordered subsets expectation maximization algorithm.

### 2.3 Image interpretation and parametric quantification

PET/CT images were analyzed using parameters quantified on a Syngo MI Workplace software platform (Siemens Healthcare). The presence of positive PLN and/or PALN was investigated. The PET voxel standardized uptake value (SUV) was calculated by normalizing the image‐derived radioactivity concentration to the whole‐body concentration (i.e., injected dose divided by patient body weight). SUV_max_ was defined as the maximum SUV in the volume of interest (VOI). MTV was defined as the volume of estimated tumor voxels with increased radioactivity uptake. The VOIs for the primary tumor and positive lymph nodes were manually drawn on the images displayed on the workstation; SUV_max_ was recorded for each VOI. The MTV of the cervical tumor was measured using a fixed boundary SUV of 3.0. Nodal SUV_max_ was defined as the maximum SUV_max_ of all positive PLNs and PALNs.

### 2.4 Systemic CCRT regimens

CCRT was administered as described previously.^4^ In brief, external‐beam radiotherapy was delivered to the whole pelvis with a radiation dose of 45 Gy. Irradiation fields were extended to the abdominal para‐aortic region if necessary. Patients may undergo brachytherapy with six fractions of 4.3 Gy delivered to point A or intensity‐modulated radiotherapy. The primary tumor and nodal lesions were treated up to a total dose of 72 Gy and 57.6 Gy, respectively. Latest techniques, such as image‐guided brachytherapy, were not used. In cohort 1, systemic therapy consisted of weekly cisplatin (40 mg/m^2^) either with or without (1:1 randomization) gemcitabine (125 mg/m^2^) administered during the course of radiotherapy lasting up to 6 cycles. In cohort 2, systemic therapy consisted of single‐agent cisplatin or platinum‐based doublet/triplet combinations. The “single agent” protocol involved weekly administration of cisplatin (40 mg/m^2^) during radiotherapy. Diverse protocols were used for the “combination regimen.” In general, cisplatin or carboplatin was used as the backbone. The drug was selected mainly on the basis of the attending physician's preference and the patient's clinical condition. The concurrent therapy began during the first week of radiation. The single‐agent cisplatin was administered every week for 6 weeks. The combination regimens were generally administered for up to six cycles. In the absence of disease progression, durvalumab, pembrolizumab, and bevacizumab were administered after CCRT.

### 2.5 Follow‐up and survival analyses

Follow‐up examinations were performed every month for 3 months after the completion of CCRT. Further follow‐up examinations were arranged every 3 months for the first 2 years, every 4 months for the third year, and every 6 months thereafter. In addition to physical examinations, the levels of serum tumor markers, including SCC antigen and carcinoembryonic antigen, were routinely measured. Imaging studies were arranged every 3–6 months for 2 years or whenever relapse was suspected. Biopsy or further imaging correlation was arranged for patients exhibiting positive or suspicious results. Cancer‐specific survival (CSS) was calculated from the date of pretreatment PET/CT to that of death from cervical cancer or censored to the date of the most recent follow‐up or death unrelated to cervical cancer. PFS was calculated from the date of pretreatment PET/CT to that of an examination indicating cancer progression/recurrence or censored to the same date as that for CSS.

### 2.6 Development of a scoring prototype for risk stratification

To determine CSS and PFS, we analyzed available data regarding clinical and imaging predictors, including age, 2009 FIGO stage (I–II vs. III–IVA), tumor grade, PLN status, PALN status, primary tumor SUV_max_, primary tumor MTV, and nodal SUV_max_. The effect of systemic therapy protocol (single agent vs. combination regimen) was also evaluated. Independent factors that were found to be significant in multivariate analysis performed to identify the predictors of CSS were used for developing a scoring prototype for risk stratification, with each independent predictor assigned a risk score of 0 or 1. This scoring prototype was used to classify the patients according to their cumulative risk scores. Next, survival curves were generated for patients with different cumulative scores.

### 2.7 Comparison between patients receiving systemic therapy with single agent or combination regimen

Because the number of patients receiving advanced combination regimens in cohort 2 was small, we performed propensity score matching (PSM) to compare the survival between the patients who received CCRT with standard single‐agent chemotherapy and those who received CCRT with combination regimens. A subset of patients receiving CCRT with single‐agent chemotherapy was constructed by selecting patients exhibiting characteristics similar to those of patients receiving combination regimens.

### 2.8 Statistical analysis

Continuous data were analyzed using the Student's *t* test or Mann–Whitney *U* test, whereas categorical data were analyzed using the Fisher's exact test or chi‐squared test. Due to the presence of both continuous and categorical predictors, univariate Cox regression analysis was first conducted to identify the significant predictors of CSS. Kaplan–Meier survival analysis and the log‐rank test were used for assessing 5‐year CSS and PFS, with a tree‐based method used for determining the optimal cutoff values for significant continuous variables for CSS.^16^ Multivariate and stepwise Cox regression analysis was then performed for identifying the independent predictors of survival. To evaluate the systemic therapy impact, patients receiving single‐agent cisplatin were matched (2:1) with those receiving combination therapy through multivariate logistic regression.^17^ All statistical analyses were performed using SAS (version 9.4; SAS Institute Inc.) and R (version 3.6.1).

## 3 RESULTS

### 3.1 Characteristics of the study cohorts

**FIGURE 1:**
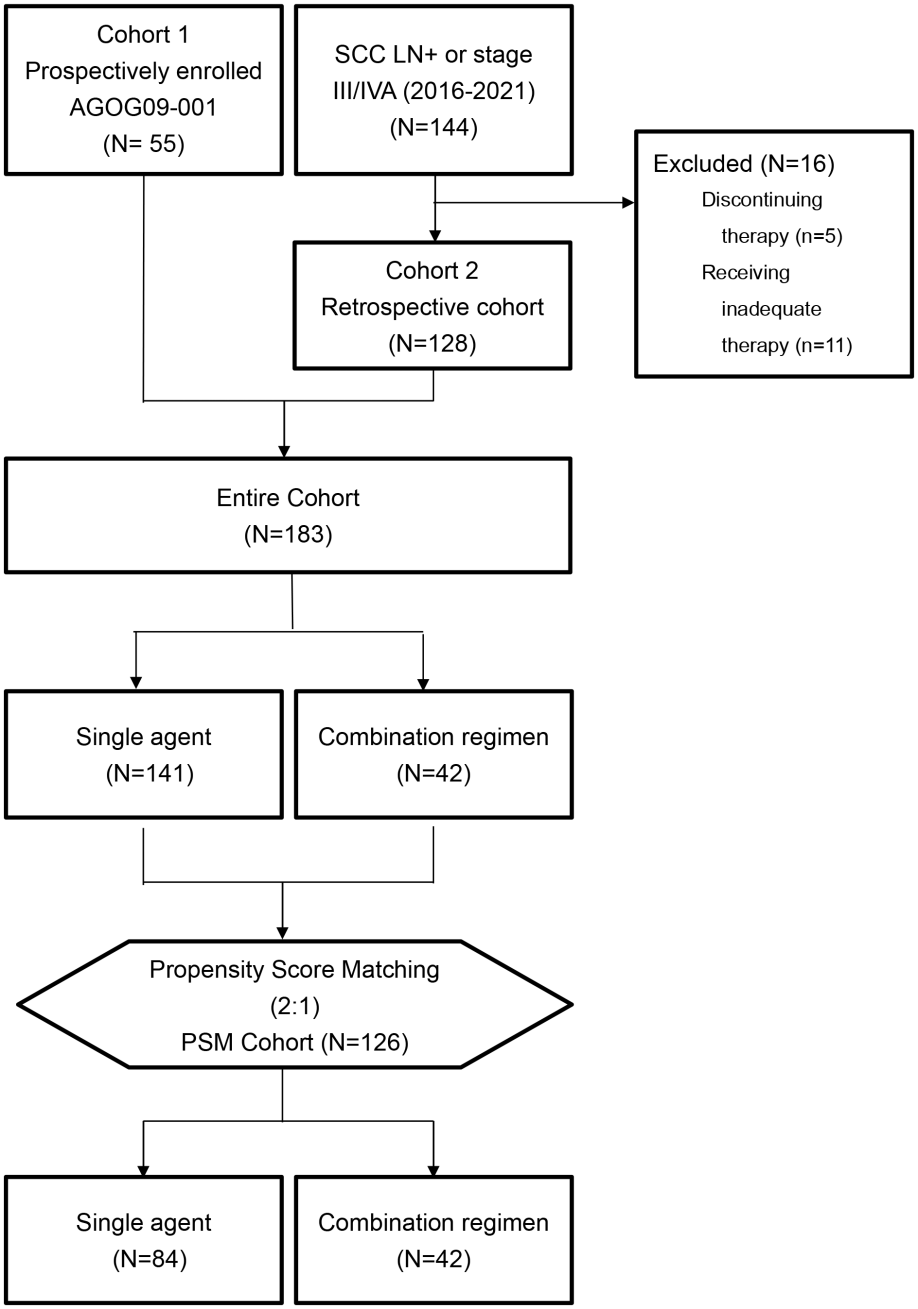
Flow diagram depicting the selection of patients for the study cohorts and PSM subset.

Cohort 1 comprised 55 patients, whereas 144 patients were retrospectively enlisted in cohort 2. From cohort 2, 5, and 11, patients were excluded because of treatment discontinuation and inadequate therapy, respectively. Figure 1 presents a flow diagram of depicting the selection of patients for the study cohorts and PSM subset. Table 1 presents the patients' baseline clinical characteristics, including their age, 2009 FIGO stage, tumor grade, PLN status, PALN status, systemic therapy for CCRT, and PET parameters. Cohorts 1 and 2 varied significantly in terms of age distributions, PLN status, systemic therapy for CCRT, and primary tumor SUV_max_.

**TABLE 1:** Clinical characteristics of the study cohorts.

Characteristic	Entire cohort			*p* Value^a^
	Combined cohort	Cohort 1	Cohort 2	
	(*N* = 183)	(*N* = 55)	(*N* = 128)	
Age, years				0.002
≤35	3 (1.6)	0 (0)	3 (2.3)	
>35 and ≤ 45	34 (18.6)	6 (10.9)	28 (21.9)	
>45 and ≤ 57	67 (36.6)	26 (47.3)	41 (32.0)	
>57 and ≤ 70	59 (32.2)	23 (41.8)	36 (28.1)	
>70 and ≤ 80	14 (7.7)	0 (0)	14 (10.9)	
>80	6 (3.3)	0 (0)	6 (4.7)	
FIGO stage (2009)				0.058
I	19 (10.4)	4 (7.3)	15 (11.7))	
II	72 (39.3)	28 (50.9)	44 (34.4)	
III	83 (45.4)	23 (41.8)	60 (46.9)	
IVA	9 (4.9)	0 (0)	9 (7.0)	
Tumor grade				0.118
MD	109 (60)	28 (51)	81 (63)	
PD	74 (40)	27 (49)	47 (37)	
PLN status				0.032
Negative	14 (7.7)	8 (14.5)	6 (4.7)	
Positive	169 (92.3)	47 (85.5)	122 (95.3)	
PALN status				0.530
Negative	134 (73.2)	42 (76.4)	92 (71.9)	
Positive	49 (26.8)	13 (23.6)	36 (28.1)	
Systemic therapy for CCRT				<0.001
Single agent	141 (77.0)	24 (43.6)	117 (91.4)	
Combination regimen	42 (23.0)	31 (56.4)	11 (8.6)	
PET parameters (mean ± SD)				
Primary tumor SUV_max_	18.2 ± 8.2	13.9 ± 5.1	20.0 ± 8.5	<0.001
Primary tumor MTV	101.6 ± 93	86.3 ± 81.4	108.2 ± 97.2	0.144
Nodal SUV_max_	7.5 ± 5.9	6.6 ± 4.3	7.9 ± 6.4	0.130

Abbreviations: FIGO, International Federation of Gynecology and Obstetrics; MD, moderately differentiated; MTV, metabolic tumor volume; PALN, para‐aortic lymph node; PD, poorly differentiated; PET, positron emission tomography; PLN, pelvic lymph node; SUV_max_, maximum standardized uptake value.

^a^ *p* values for the difference between cohorts 1 and 2; between‐cohort comparison was performed using the chi‐square or Fisher's exact test.

### 3.2 Findings related to follow‐up and survival

The median follow‐up duration for the combined cohort was 51 (range, 2–164) months. A total of 49 patients died, of whom 2 died for reasons unrelated to cervical cancer. Cancer progression or recurrence occurred in 58 patients. In the combined cohort, the rates of 5‐year PFS and CSS were 66% and 69%, respectively (Figure S1). In the AGOG09‐001 trial, the 5‐year PFS and CSS rates were 65% and 70%, respectively (Figure S2); the median follow‐up duration was 119 (range, 4–164) months.

### 3.3 Determination of significant predictors and cutoff values

The results of the univariate Cox regression analysis are presented in Table 2. Age, FIGO stage, PALN status, and primary tumor MTV were significant predictors of CSS and selected for further analyses. The 5‐year CSS and PFS rates assessed by Kaplan–Meier analysis and the optimal cutoff values for continuous variables are presented in Table 3. All the selected predictors remain significant, and the optimal cutoff values for age and primary tumor MTV are 66 years and 87 mL, respectively. The statistical results of systemic therapy protocol for survival were also appended for reference.

**TABLE 2:** Results of the univariate Cox regression analysis for CSS and PFS in the combined cohort.

Characteristic	CSS		PFS	
	HR	*p* Value	HR	*p* Value
Age	1.043	<0.001	1.031	0.004
FIGO stage (III–IVA vs. I–II)	2.006	0.022	1.678	0.054
Tumor grade (PD vs. MD)	1.502	0.163	1.555	0.093
PLN status (+ vs. −)	1.573	0.449	1.294	0.619
PALN status (+ vs. −)	2.567	0.001	2.914	<0.001
Primary tumor SUV_max_	1.004	0.847	0.997	0.879
Primary tumor MTV	1.003	0.002	1.004	<0.001
Nodal SUV_max_	1.036	0.122	1.034	0.099

Abbreviations: CSS, cancer‐specific survival; FIGO, International Federation of Gynecology and Obstetrics; HR, hazard ratio; MD, moderately differentiated; MTV, metabolic tumor volume; PALN, para‐aortic lymph node; PD, poorly differentiated; PFS, progression‐free survival; PLN, pelvic lymph node; SUV_max_, maximum standardized uptake value.

**TABLE 3:** Results of the Kaplan–Meier analysis for CSS and PFS in the combined cohort.

	CSS			PFS		
Characteristic	Cutoff	5‐year rate (%)	*p* Value	Cutoff	5‐year rate (%)	*p* Value
Age (≥ vs. <)	66	38.1, 74.8	<0.001	58	52.7, 76.6	<0.001
FIGO stage (III–IVA vs. I–II)		57.7, 79.6	0.020		57.9, 74.6	0.052
PALN status (+ vs. −)		48.8, 75.6	0.001		40.1, 75.2	<0.001
Primary tumor MTV (≥ vs. <)	87	45.5, 83.2	<0.001	87	43.0, 81.5	<0.001
Systemic treatment (C vs. S)		73.2, 67.5	0.564		72.1, 64.8	0.483

Abbreviations: C, combination regimen; CSS, cancer‐specific survival; FIGO, International Federation of Gynecology and Obstetrics; MTV, metabolic tumor volume; PALN, para‐aortic lymph node; PFS, progression‐free survival; S, single agent.

### 3.4 Results of univariate and multivariate Cox regression analyses

The results of the Cox regression analysis performed to identify the predictors of CSS are presented in Table 4. In the univariable analysis, age ≥ 66 years, FIGO stage III‐IVA, positive PALN, and primary tumor MTV ≥87 mL were revealed to be significant predictors. In the multivariate analysis, FIGO stage was found to be a nonsignificant predictor. Stepwise Cox regression revealed that the most significant predictor was age (*p* < 0.001), followed by primary tumor MTV (*p* = 0.014) and PALN status (*p* = 0.027). The results of Cox regression analyses for PFS are presented in Table 5. The most significant predictor was age (*p* = 0.001), followed by primary tumor MTV (*p* = 0.004) and PALN status (*p* = 0.004).

**TABLE 4:** Results of the Cox regression analysis for CSS in the combined cohort.

Characteristics	*n*	Univariate analysis			Multivariate analysis			Stepwise analysis (forward)		
		HR	95% CI	*p* Value	HR	95% CI	*p* Value	HR	95% CI	*p* Value
Age, years										
<66	150	1 (ref)			1 (ref)			1 (ref)		
≥66	33	2.993	1.634–5.480	<0.001	3.036	1.589–5.804	0.001	3.262	1.739–6.116	<0.001
FIGO stage										
I, II	91	1 (ref)			1 (ref)					
III, IVA	92	2.006	1.105–3.641	0.022	1.504	0.752–3.008	0.248			
PALN status										
Negative	134	1 (ref)			1 (ref)			1 (ref)		
Positive	49	2.565	1.433–4.589	0.001	2.240	1.188–4.222	0.013	2.036	1.084–3.826	0.027
Primary tumor MTV										
<87 mL	109	1 (ref)			1 (ref)			1 (ref)		
≥87 mL	74	3.272	1.799–5.952	<0.001	2.119	1.006–4.463	0.048	2.245	1.178–4.276	0.014

Abbreviations: CSS, cancer‐specific survival; FIGO, International Federation of Gynecology and Obstetrics; MTV, metabolic tumor volume; PALN, para‐aortic lymph node.

**TABLE 5:** Results of the Cox regression analysis for PFS in the combined cohort.

Characteristics	*n*	Univariate analysis			Multivariate analysis			Stepwise analysis (forward)		
		HR	95% CI	*p* Value	HR	95% CI	*p* Value	HR	95% CI	*p* Value
Age, years										
<66	150	1 (ref)			1 (ref)			1 (ref)		
≥66	33	2.357	1.338–4.155	0.003	2.555	1.394–4.683	0.002	2.774	1.534–5.019	0.001
FIGO stage										
I, II	91	1 (ref)			1 (ref)					
III, IVA	92	1.677	0.990–2.841	0.055	1.045	0.573–1.906	0.885			
PALN status										
Negative	134	1 (ref)			1 (ref)			1 (ref)		
Positive	49	2.911	1.728–4.904	<0.001	2.528	1.424–4.488	0.002	2.338	1.321–4.138	0.004
Primary tumor MTV										
<87 mL	109	1 (ref)			1 (ref)			1 (ref)		
≥87 mL	74	3.690	2.139–6.366	<0.001	2.652	1.380–5.097	0.003	2.360	1.308–4.259	0.004

Abbreviations: FIGO, International Federation of Gynecology and Obstetrics; MTV, metabolic tumor volume; PALN, para‐aortic lymph node; PFS, progression‐free survival.

### 3.5 Scoring prototype for prognostic stratification

**FIGURE 2:**
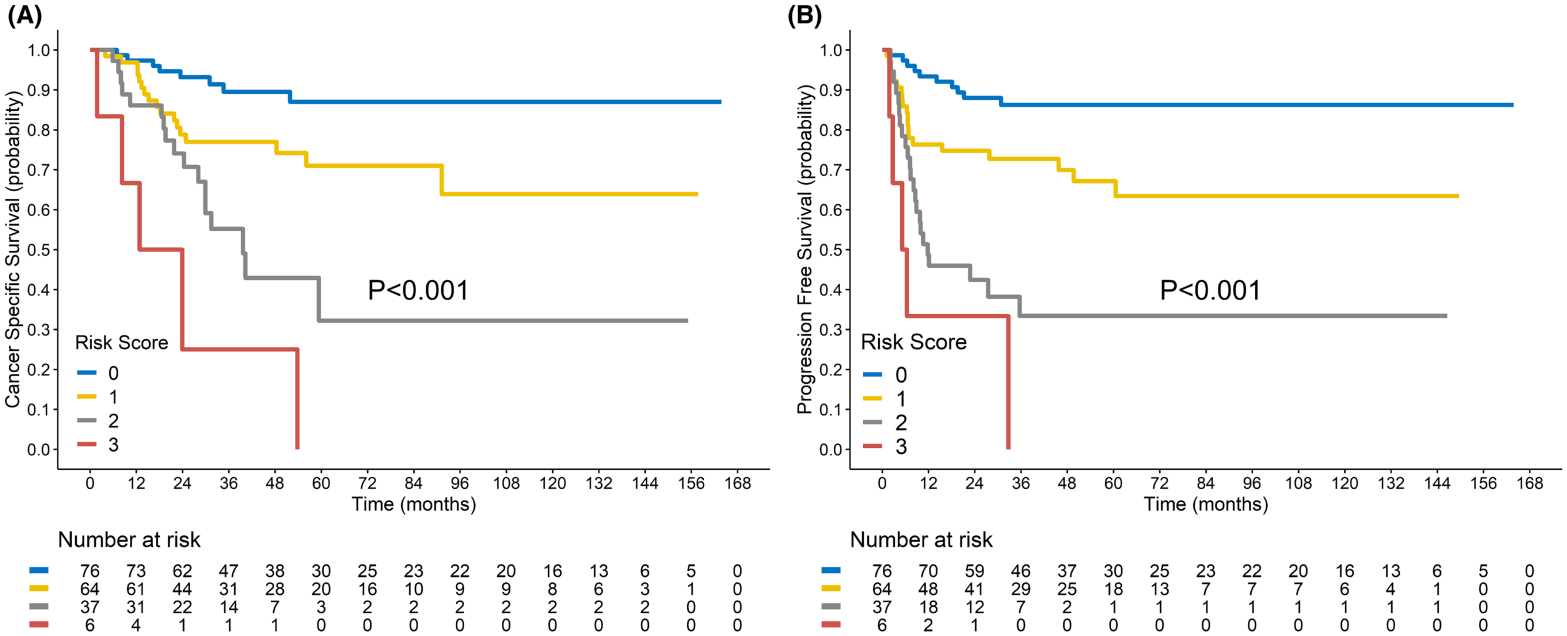
Survival curves for patients with different cumulative risk scores. (A) Cancer‐specific survival. (B) Progression‐free survival.

**FIGURE 3:**
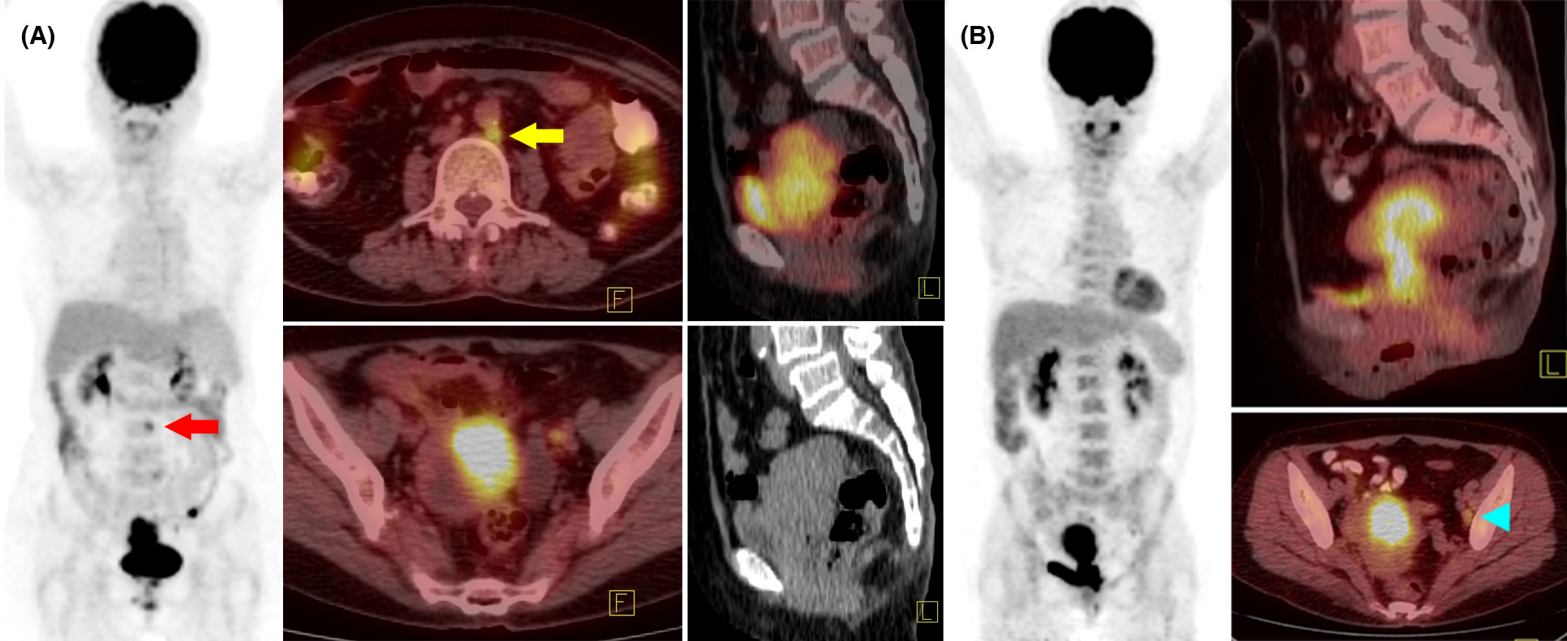
Representative cases of different survival rates in patients with different cumulative risk scores. (A) This is the case of a 79‐year‐old patient with FIGO 2009 stage IIIB cervical cancer. Pretreatment PET/CT revealed a bulky primary tumor (SUV_max_ = 14.8; MTV = 94 mL) with small positive pelvic and para‐aortic nodes (arrows). This patient had a cumulative risk score of 3 and received CCRT with cisplatin and bevacizumab. However, PET/CT imaging performed 3 months after the completion of CCRT revealed supraclavicular nodal metastases; nevertheless, cervical tumor and pelvic/para‐aortic lymph nodes indicated prominent regression. The patient died after another 3 months. (B) This is the case of a 57‐year‐old patient with FIGO stage IIIB cervical cancer. Pretreatment PET/CT revealed a cervical tumor with parametrial extension (SUV_max_ = 13.9; MTV = 85 mL) and a positive left external iliac lymph node (arrow head). This patient had a cumulative risk score of 0 and received standard CCRT with weekly cisplatin. The patient remained disease‐free for 3 years as of the latest follow‐up.

On the basis of the aforementioned results, the following factors were selected for use in the development of the scoring prototype for risk stratification: age ≥ 66 years, primary tumor MTV ≥87 mL, and positive PALN; each factor was assigned a score of 1. Cumulative risk scores of 0, 1, 2, and 3 corresponded to 5‐year PFS and CSS rates of 86.2% and 86.9%, 67.1% and 71.0%, 33.4% and 32.2%, and 0% and 0%, respectively. The survival curves for patients with different cumulative risk scores varied significantly (*p* < 0.001; Figure 2). Two representative cases are presented in Figure 3.

### 3.6 Comparison between patients receiving systemic therapy with single agent and combination regimen

In cohort 1, 31 patients received cisplatin with gemcitabine, and 24 patients received cisplatin only. In cohort 2, 117 patients received weekly cisplatin or carboplatin, and 11 patients received combination therapy. The combination regimens were as follows: cisplatin‐topotecan, carboplatin‐topotecan, cisplatin‐topotecan‐pembrolizumab, cisplatin‐bevacizumab, cisplatin‐nivolumab, cisplatin‐durvalumab, cisplatin‐pembrolizumab, and cisplatin‐bevacizumab‐pembrolizumab. The baseline characteristics of the patients receiving combination therapy are presented in Table S1. The patients receiving combination therapy in cohort 2 were significantly older and had a higher rate of positive PALN than those in cohort 1. Two PET parameters, primary tumor SUV_max_ and nodal SUV_max_, were also significantly different.

**FIGURE 4:**
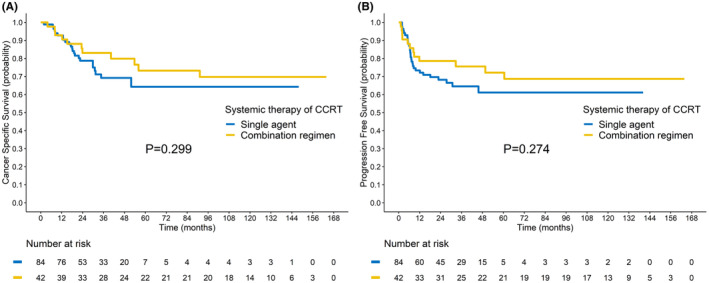
Survival curves for propensity score‐matched patients receiving single‐agent concurrent chemoradiotherapy or combination therapy. (A) Cancer‐free survival. (B) Progression‐free survival.

Two matching variables, FIGO stage and PALN status, were employed to reduce the effects of confounding during PSM subset selection. The PSM cohort comprised 126 patients; their baseline characteristics are summarized in Table S2. No significant differences were noted between patients receiving CCRT with single‐agent chemotherapy and combination regimens except for the primary tumor SUV_max_. The corresponding survival curves are presented in Figure 4. Survival rates did not vary significantly different between these two PSM groups (CSS, *p* = 0.299; PFS, *p* = 0.274); nevertheless, the combination regimens tended to confer mild survival benefits. The comparison between CCRT with single agent versus combination regimen in the combined cohort revealed no significant difference (Figure S3). In the AGOG09‐001 trial, no significant difference was noted in PFS and CSS between the treatment arms, even after extended follow‐up (Figure S4). For patients with different cumulative risk scores in the combined cohort, the comparison between CCRT with single agent and combination regimen is presented in Figures S5–S8, respectively. In the patients with cumulative risk scores 2, the benefit of combination regimen approached borderline significance.

## 4 DISCUSSION

In the meta‐analysis of LACC without distant metastasis, CCRT compared with radiotherapy only was found to confer a 6% benefit in terms of OS.^2^ Post‐CCRT adjuvant chemotherapy with carboplatin and paclitaxel was investigated in the OUTBACK trial, but no obvious survival benefit was discovered.^5^ In the CALLA trial, durvalumab or placebo was added to cisplatin during CCRT and post‐CCRT maintenance therapies. At a median follow‐up of 18.5 months, durvalumab group did not show a statistically significant improvement in PFS (HR 0.84, *p* = 0.174).^6^ Use of platinum doublet regimens as neoadjuvant chemotherapy before CCRT has also been attempted with controversial results.^18^, ^19^ In the randomized INTERLACE trial, weekly carboplatin‐paclitaxel was employed in the neoadjuvant setting with pending results (ClinicalTrials.gov identifier: NCT01566240).^20^ More intricate prognostic predictors are required for the effective identification of patients for whom standard CCRT may be inadequate. To identify such predictors, we conducted the present study. Our previous trials and the present study included only patients with SCC.^4^, ^14^ This is because patients with adenocarcinoma or adenosquamous carcinoma generally have significantly worse outcomes than do patients with SCC, regardless of whether they undergo primary radical surgery or primary radiotherapy.^21^, ^22^

A study was conducted using 8538 patients aged ≥50 years who were selected from the Surveillance, Epidemiology, and End Results database. The results of the study reveal that age at diagnosis significantly affected disease prognosis.^23^ Compared with the risk of adverse outcomes in patients aged 50–60 years, the HR for patients aged 60–70 years was 1.229, and that for those aged >70 years was 1.625. Older patients often have degenerative or chronic diseases; these patients may exhibit poor tolerance to surgery and experience serious side effects following therapy. Complications in older patients are more difficult to manage and thus result in poor prognosis and higher mortality rates. The Kaplan–Meier analysis performed in our study revealed the age of ≥66 years to be the optimal cutoff age for predicting adverse CSS; the corresponding HR derived from the stepwise Cox regression analysis was 3.262. However, a study reported that patients aged ≤30 years have poor prognosis.^24^ In the present study, patients aged <35 years had been excluded in cohort 1 and there were only three patients aged <35 years in cohort 2; two patients aged <30 years remained disease‐free with a follow‐up duration of 49 and 67 months, respectively, and another patient aged 32 remained disease‐free with a follow‐up duration of 29 month.

Metabolic tumor volume estimated by PET imaging, represents a 3‐dimensional value and can be easily measured. Chung et al.^25^ identified MTV to be a significant prognostic factor for survival in 63 patients with a FIGO stage ranging from IB to IIA before radical hysterectomy. We previously reported a significant association between pretreatment primary tumor MTV and local failure and poor OS.^14^ In a retrospective analysis of 508 patients with radically treated cervical cancer, primary tumor MTV, but not primary tumor SUV_max_, was discovered to be a significant factor influencing survival.^26^ This finding corroborates that of our study.

Para‐aortic lymph node metastasis is a strong predictor of poor prognosis. The incorporation of nodal involvement in the FIGO 2018 staging definition for cervical cancer highlighted the need for assessing PALN status.^27^ In a study involving 618 patients with locally advanced cervical SCC, PALN metastasis was found to be an independent prognostic factor for PFS and OS.^28^ In a study involving patients with PALN metastasis, SUV_max_ of the involved PALN metastasis and nodal volume or total lesion glycolysis were used to stratify patients in terms of survival.^29^

In our study, the rates of 5‐year PFS and CSS in the combined cohort were 66% and 69%, respectively. Age (≥ 66 years), MTV (≥ 87 mL), and PET/CT‐defined PALN metastasis were found to be independent predictors of poor survival outcomes. On the basis of these risk predictors, the patients were assigned a cumulative risk score of 0–3. We recommend that patients with a cumulative risk score of 0 receive CCRT with single‐agent cisplatin and that for others, novel combination therapies or clinical trials be considered.

We performed PSM to compare the survival between patients receiving CCRT with a single agent and those receiving combination regimen. In cohort 1, patients were randomly selected for combination therapy. By contrast, in cohort 2, only 11 patients received combination therapy. Because of the limited sample size of the PSM cohort, the survival difference between the two treatment groups did not achieve statistical significance. However, a mild trend favoring the combination regimen was noted.

The strengths of the present study are as follows: we included a sufficient number of patients who received adequate treatment for LACC and underwent pretreatment ^18^F‐FDG PET/CT for analysis, and we unveiled the independently significant predictors of poor survival outcomes in this patient population. Our study has some limitations. First, both perspective and retrospective data were analyzed. Second, data were obtained from a single center and from patients belonging to a single ethnicity, which limits the generalizability of our findings. Third, we lacked information regarding biological markers. Finally, the number of patients receiving combination regimens was small in cohort 2, and the regiments were heterogeneous. Nevertheless, our scoring prototype for risk stratification may guide clinicians in selecting an effective therapeutic regimen for patients with locally advanced cervical SCC.

## 5 CONCLUSIONS

A novel scoring protocol has been proposed for patients with advanced cervical SCC with FIGO stage III/IVA or nodal metastasis. Age (≥66 years), primary tumor MTV (≥87 mL), and PET/CT‐defined PALN metastasis may serve as independent predictors of poor survival outcomes. Patients with a cumulative risk score of 0 may receive single‐agent CCRT with satisfactory outcomes; these patients may be excluded from trials involving the administration of novel combination therapies. However, for patients with a cumulative risk score of ≥1, more intensive treatment or clinical trials may be considered. Validation of the present study in a large multicenter trial is warranted.

## AUTHOR CONTRIBUTIONS

**Feng‐Yuan Liu:** Conceptualization (equal); investigation (lead); methodology (equal); project administration (equal); resources (equal); writing – original draft (lead); writing – review and editing (equal). **Wei‐chun Chen:** Data curation (equal); investigation (equal); validation (equal); writing – original draft (equal). **Yu‐Bin Pan:** Formal analysis (equal); methodology (equal); software (equal); visualization (equal). **Chun‐Chieh Wang:** Funding acquisition (equal); resources (equal). **Yun‐Hsin Tang:** Resources (equal); writing – original draft (supporting). **Hung‐Hsueh Chou:** Resources (equal); writing – original draft (supporting). **Angel Chao:** Resources (equal); writing – original draft (supporting). **Lan‐Yan Yang:** Formal analysis (equal); methodology (equal); visualization (equal). **Chyong‐Huey Lai:** Conceptualization (equal); investigation (equal); methodology (equal); resources (equal); writing – original draft (lead); writing – review and editing (equal).

## CONFLICT OF INTEREST STATEMENT

The authors declare no conflicts of interest.

## Supporting information


Appendix S1.

## Data Availability

The data that support the findings of this study are available from the corresponding author upon reasonable request.
