# Supplementary material for: Clinical factor‐based risk stratification for precision therapy in locally advanced squamous cell carcinoma of the uterine cervix
Source: Cancer Med. 2024 Jan 8;13(1):e6746. doi: 10.1002/cam4.6746 (PMC10807568; doi:10.1002/cam4.6746)

Supplementary Table 1. Characteristics of patients receiving combination regimens

|  |  | **Patients receiving combination regimens** | | |  |
| --- | --- | --- | --- | --- | --- |
| **Characteristic** | | **Combined cohort** | **Cohort 1** | **Cohort 2** | ***P* value*^a^*** |
|  |  | **(N = 42)** | **(N = 31)** | **(N = 11)** |  |
| **Age, years** | |  |  |  | 0.036 |
|  | ≤35 | 1 (2.4) | 0 (0) | 1 (9.1) |  |
|  | >35and ≤45 | 7 (16.7) | 5 (16.1) | 2 (18.2) |  |
|  | >45and ≤57 | 17 (40.5) | 15 (48.4) | 2 (18.2) |  |
|  | >57 and ≤70 | 15 (35.7) | 11 (35.5) | 4 (36.4) |  |
|  | >70 and ≤80 | 2 (4.8) | 0 (0) | 2 (18.2) |  |
|  | >80 | 0 (0) | 0 (0) | 0 (0) |  |
| **FIGO stage (2009)** | |  |  |  | 0.097 |
|  | I | 3 (7.1) | 2 (6.5) | 1 (9.1) |  |
|  | II | 19 (45.2) | 16 (51.6) | 3 (27.3) |  |
|  | III | 18 (42.9) | 13 (41.9) | 5 (45.5) |  |
|  | IVA | 2 (4.8) | 0 (0) | 2 (18.2) |  |
| **PLN status** | |  |  |  | 0.554 |
|  | Negative | 3 (7.1) | 3 (9.7) | 0 (0) |  |
|  | Positive | 39 (92.9) | 28 (90.3) | 11 (100) |  |
| **PALN status** | |  |  |  | 0.002 |
|  | Negative | 28 (66.7) | 25 (80.6) | 3 (27.3) |  |
|  | Positive | 14 (33.3) | 6 (19.4) | 8 (72.7) |  |
| **Systemic therapy of combination regimens** | | |  |  | <0.001 |
|  | Combination chemotherapy | 33 (78.6) | 31 (100) | 2 (18.2) |  |
|  | Adding bevacizumab | 1 (2.4) | 0 (0) | 1 (9.1) |  |
|  | Adding IO agents | 5 (11.9) | 0 (0) | 5 (45.5) |  |
|  | Adding both bevacizumab and IO agents | 3 (7.1) | 0 (0) | 3 (27.3) |  |
| **PET parameters (mean ± SD)** | | | | |  |
|  | Primary tumor SUV_max_ | 15.2 ± 5.3 | 13.5 ± 4.4 | 19.9 ± 5 | <0.001 |
|  | Primary tumor MTV | 95.1 ± 83.8 | 90.5 ± 84.8 | 107.9 ± 83.3 | 0.560 |
|  | Nodal SUV_max_ | 7.3 ± 3.5 | 6.4 ± 2.9 | 9.4 ± 4.1 | 0.014 |

*^a^ P* values: difference between cohorts 1 and 2 by chi-square or Fisher’s exact test.

FIGO International Federation of Gynecology and Obstetrics; IO immuno-oncology; MD: moderately differentiated; MTV: metabolic tumor volume; PALN: para-aortic lymph node; PD: poorly differentiated; PLN: pelvic lymph node; SUV_max_: maximum standardized uptake value.

Supplementary Table 2. Patient characteristics of propensity score matched (2:1) cohort

|  |  |  | **Mean ± SD / Count (%)** | | |  |
| --- | --- | --- | --- | --- | --- | --- |
|  |  |  | **Single agent**  **(N=84)** |  | **Combination regimen**  **(N=42)** | **P-value** |
| **Age** |  |  | 56.1 ± 11.8 |  | 54.4 ± 10.9 | 0.435 |
| **Grade** |  |  |  |  |  | 0.120 |
|  | **2** |  | 56 (66.7) |  | 22 (52.4) |  |
|  | **3** |  | 28 (33.3) |  | 20 (47.6) |  |
| **FIGO** |  |  |  |  |  | 0.970 |
|  | **I** |  | 6 (7.1) |  | 3 (7.1) |  |
|  | **II** |  | 34 (40.5) |  | 19 (45.2) |  |
|  | **III** |  | 40 (47.6) |  | 18 (42.9) |  |
|  | **IVA** |  | 4 (4.8) |  | 2 (4.8) |  |
| **PLN** |  |  |  |  |  | 0.685 |
|  | **Negative** |  | 4 (4.8) |  | 3 (7.1) |  |
|  | **Positive** |  | 80 (95.2) |  | 39 (92.9) |  |
| **PALN** |  |  |  |  |  | >0.999 |
|  | **Negative** |  | 56 (66.7) |  | 28 (66.7) |  |
|  | **Positive** |  | 28 (33.3) |  | 14 (33.3) |  |
| **PET parameters** | |  |  |  |  |  |
|  | Primary tumor SUV_max_ |  | 20.1 ± 9.3 |  | 15.2 ± 5.3 | <0.001 |
|  | Primary tumor MTV |  | 110.9 ± 102.8 |  | 95.1 ± 83.8 | 0.389 |
|  | Nodal SUV_max_ |  | 8.1 ± 6.6 |  | 7.3 ± 3.5 | 0.373 |

We used the t-test on continuous variable and Chi-square or Fisher's exact test on categorical data

Supplementary Figure 1. Survival curves for the combined cohort (N=183). (A) Cancer-free survival. (B) Progression-free survival.

(A)


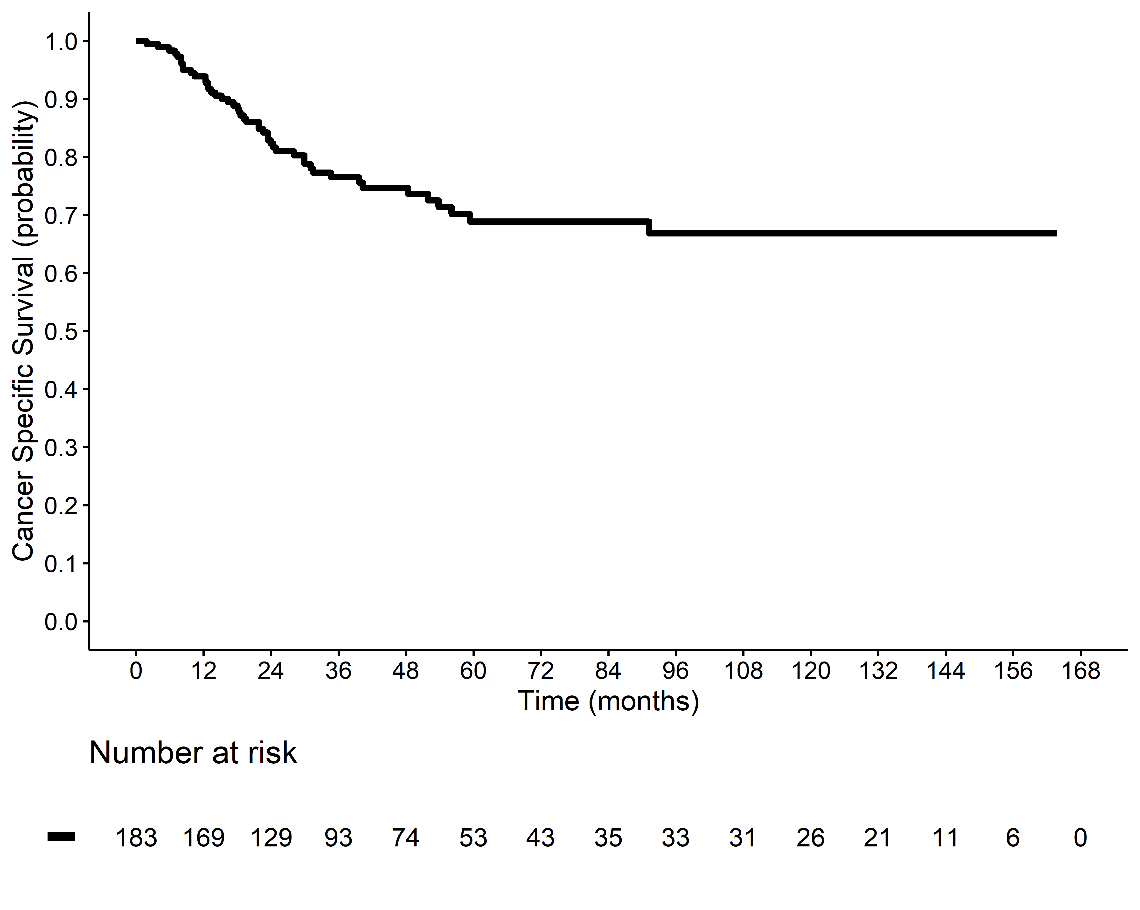


(B)


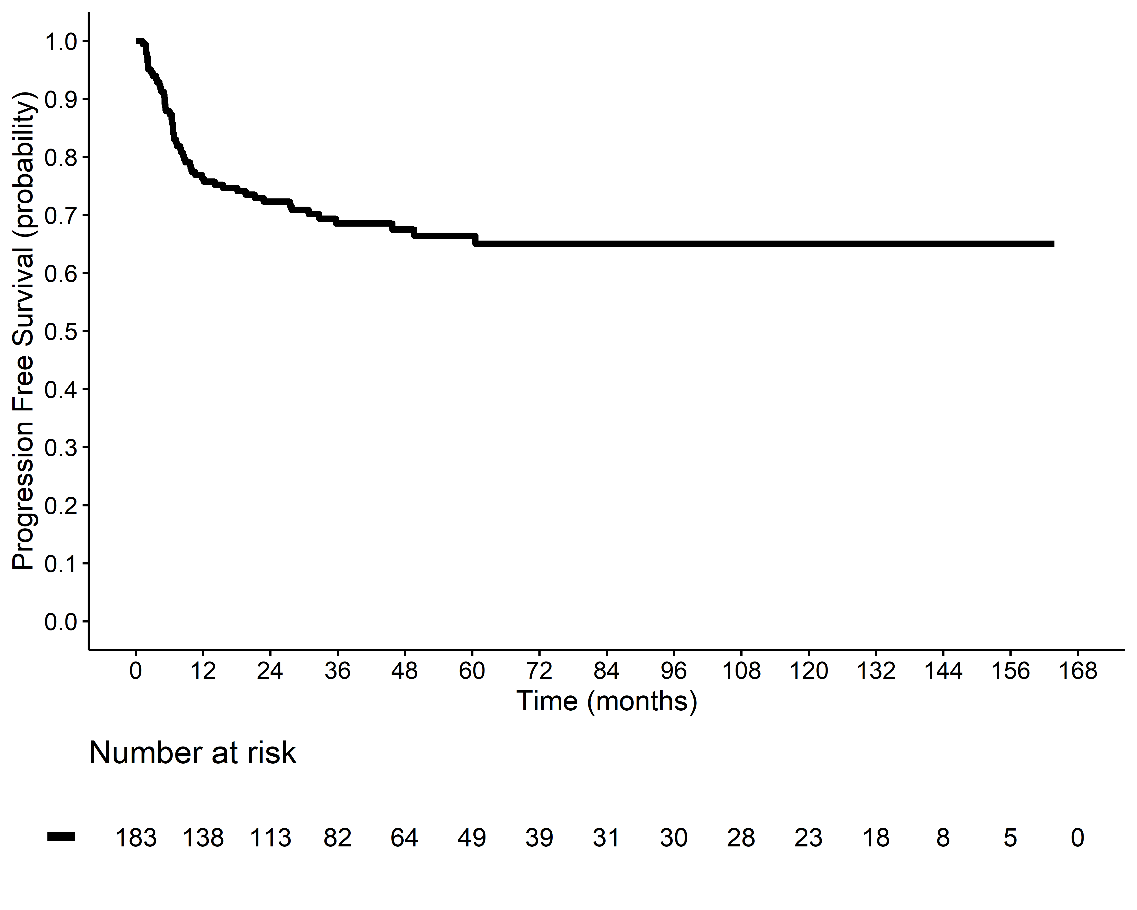


Supplementary Figure 2. Survival curves for cohort 1 (N=55). (A) Cancer-free survival. (B) Progression-free survival.

(A)


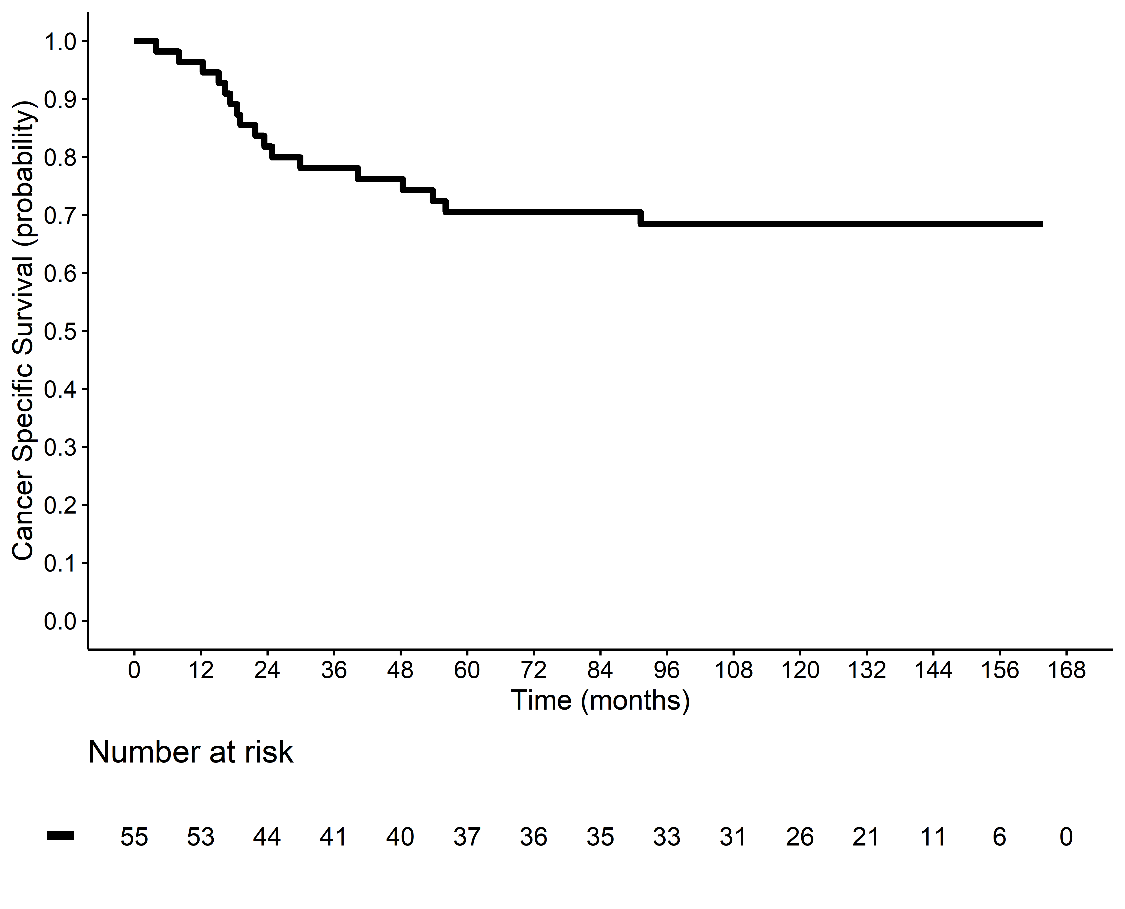


(B)


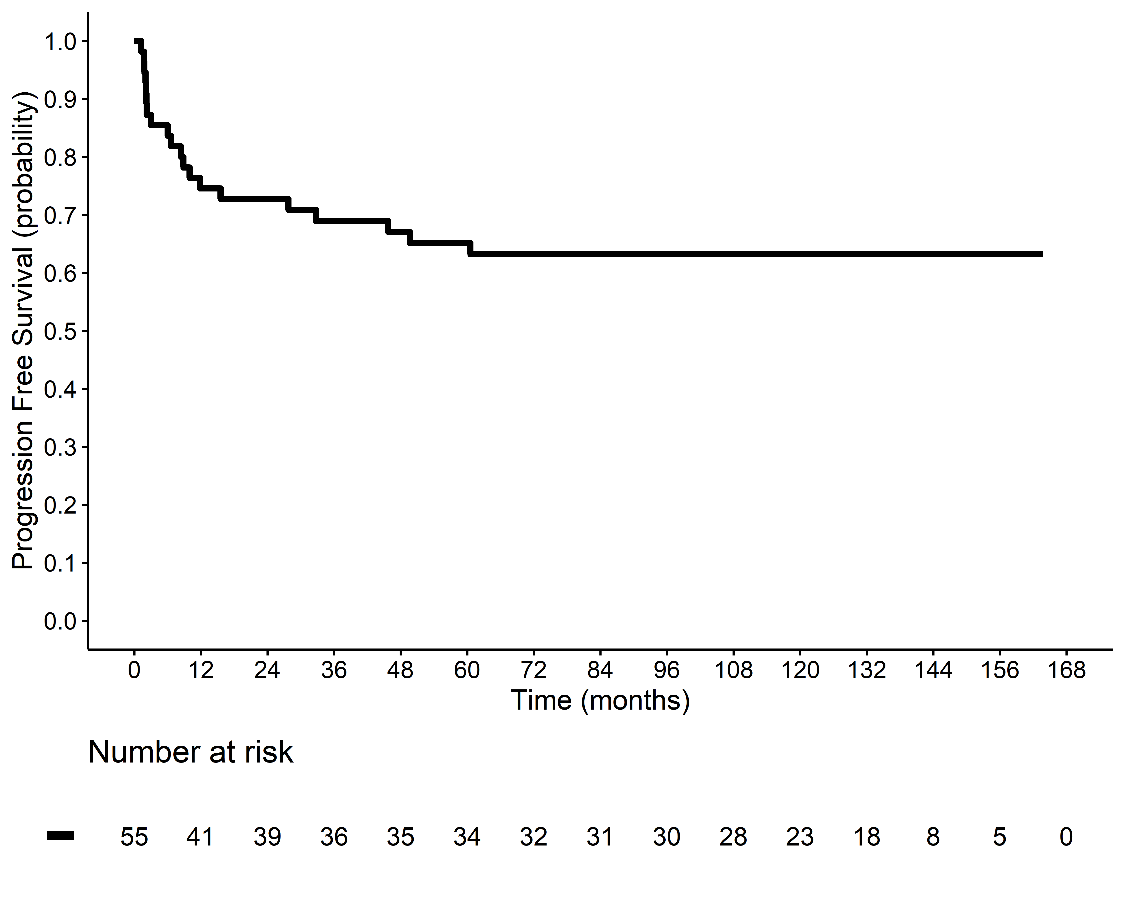


Supplementary Figure 3. Survival curves for patients receiving CCRT with single agent versus with combination therapy in the combined cohort. (A) Cancer-free survival. (B) Progression-free survival.

(A)


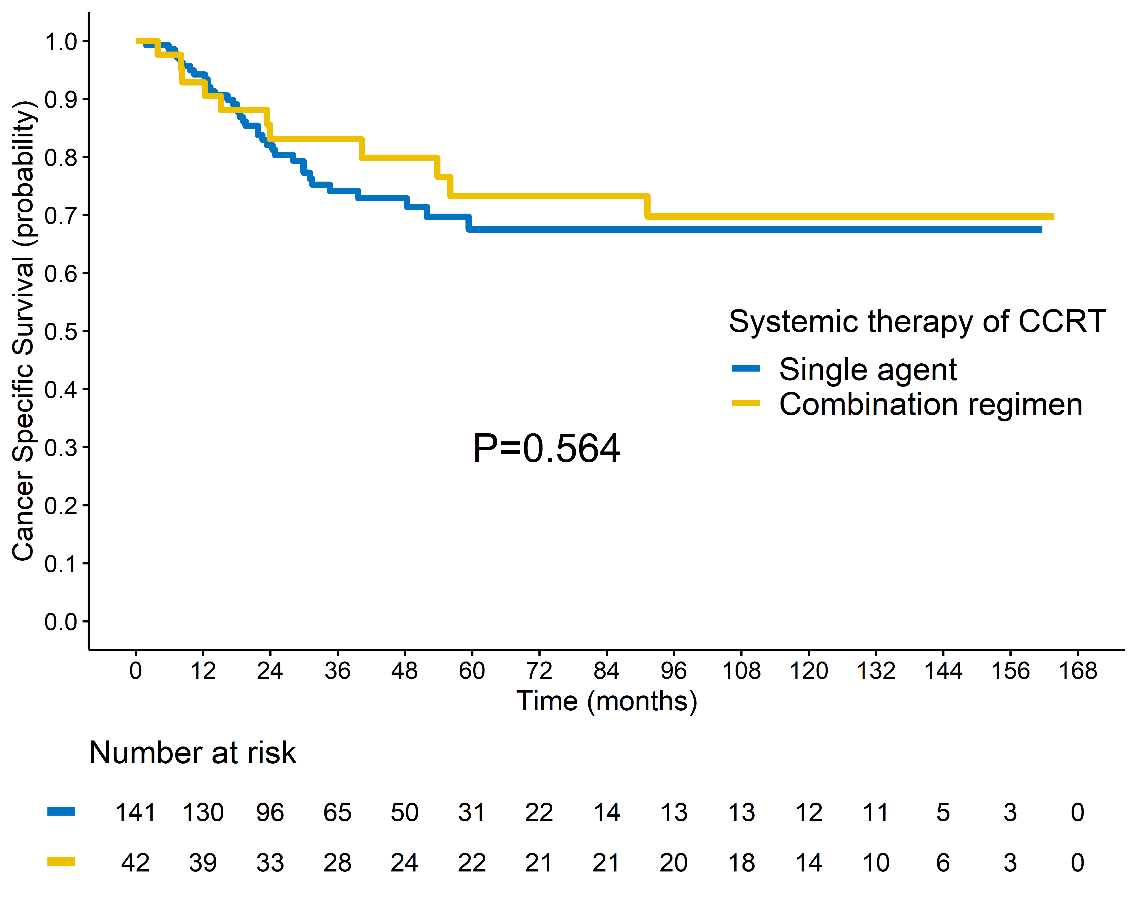


(B)


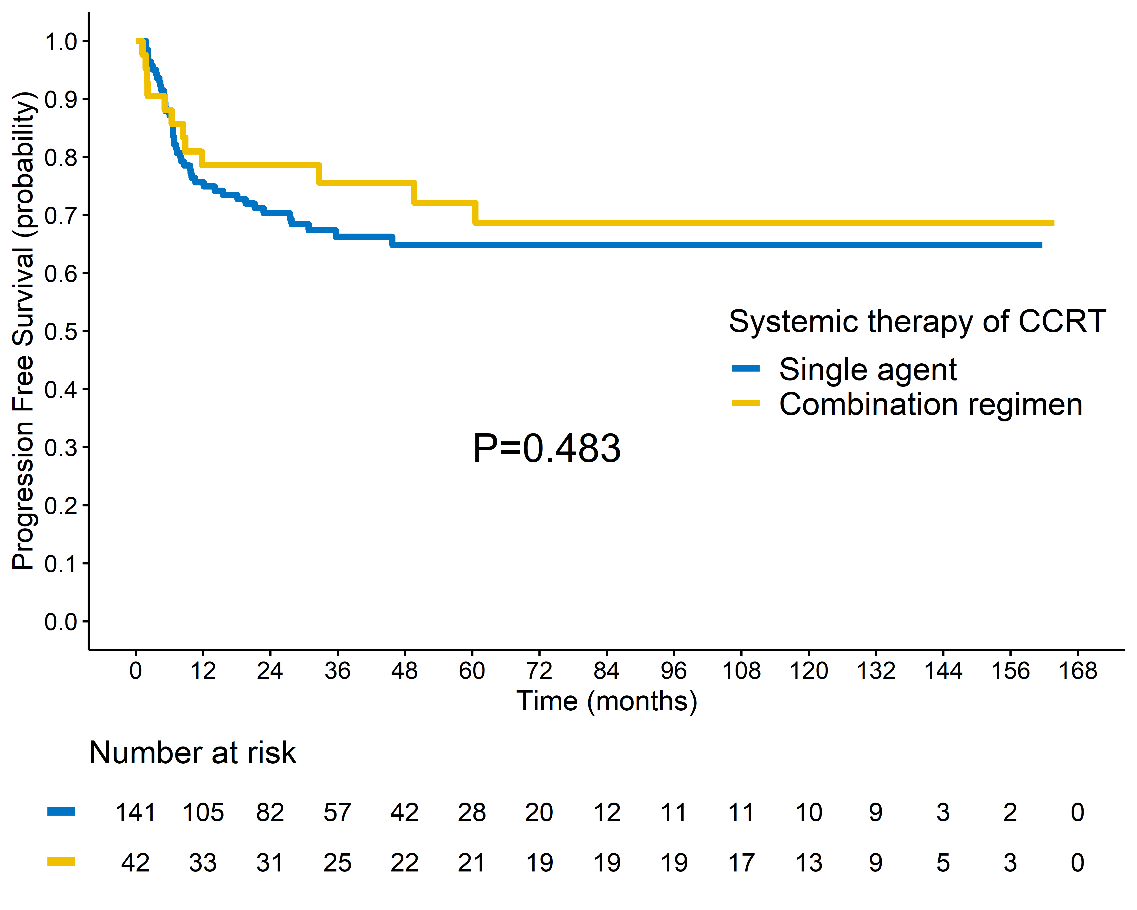


Supplementary Figure 4. Survival curves for patients receiving CCRT with single agent versus with combination regimen in cohort 1. (A) Cancer-free survival. (B) Progression-free survival.

(A)


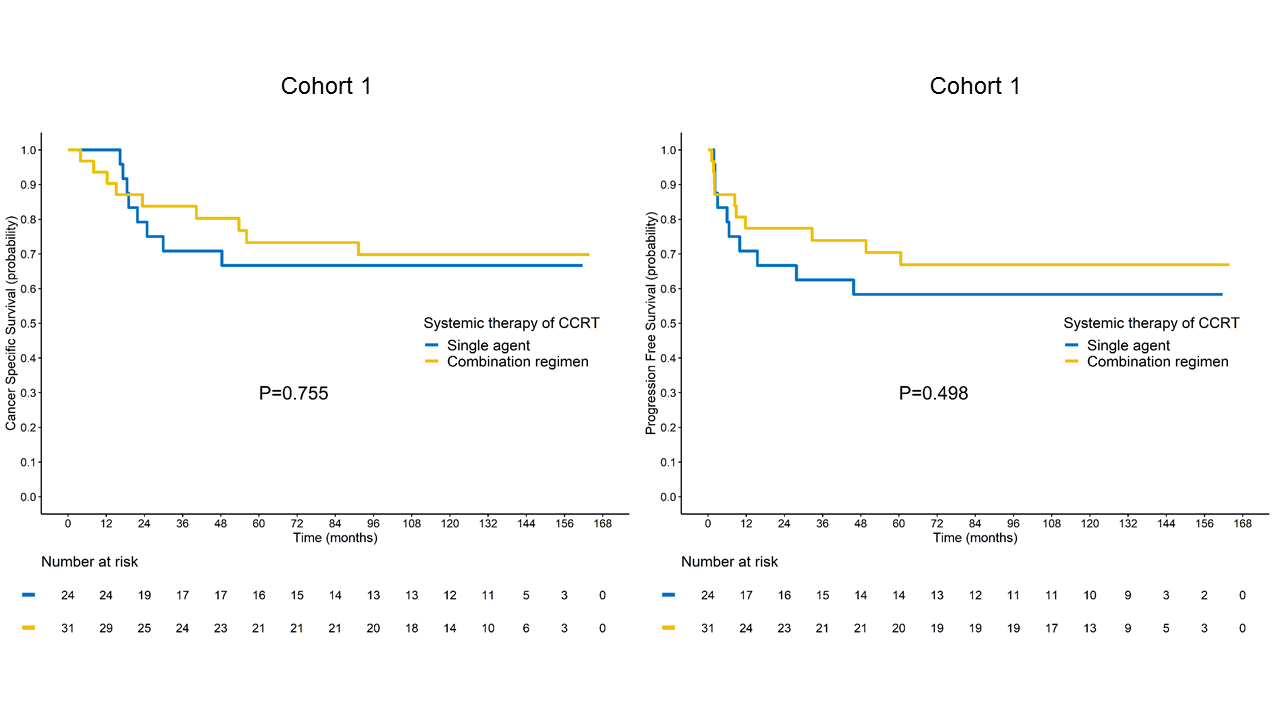


(B)


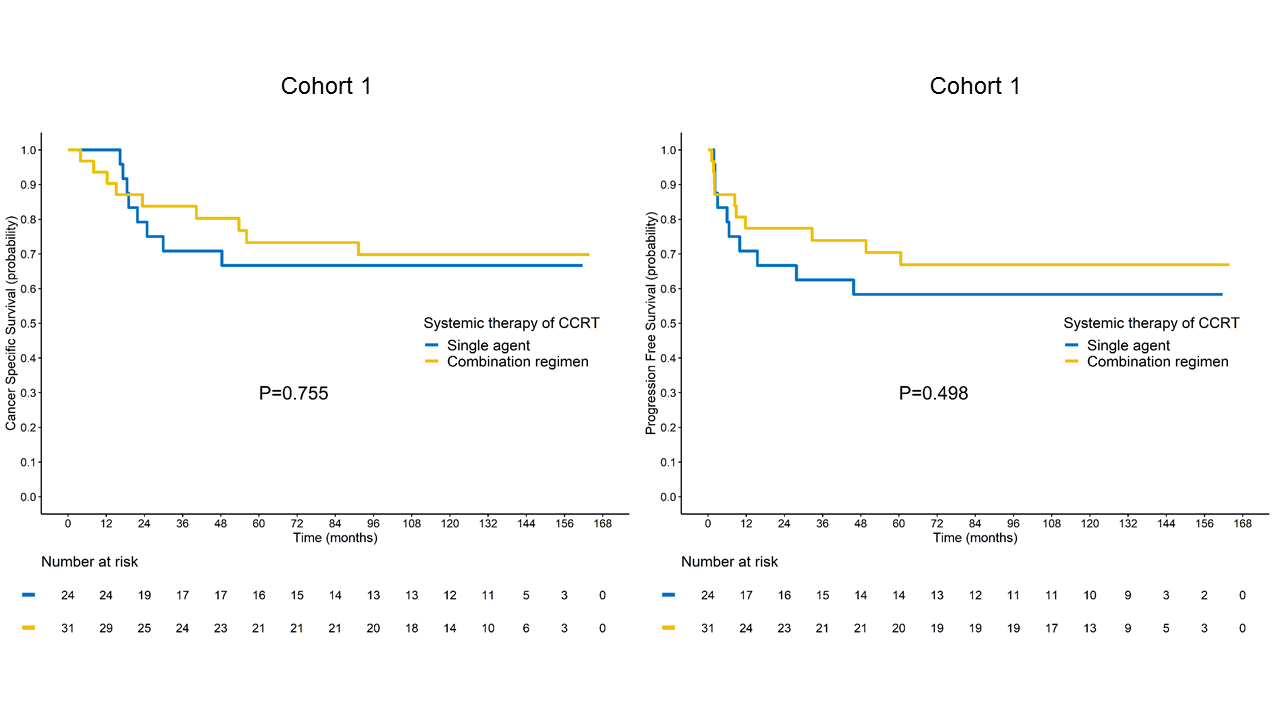


Supplementary Figure 5. Survival curves for patients receiving CCRT with single agent versus with combination therapy in the combined cohort with cumulative risk score 0. (A) Cancer-free survival. (B) Progression-free survival.

(A)


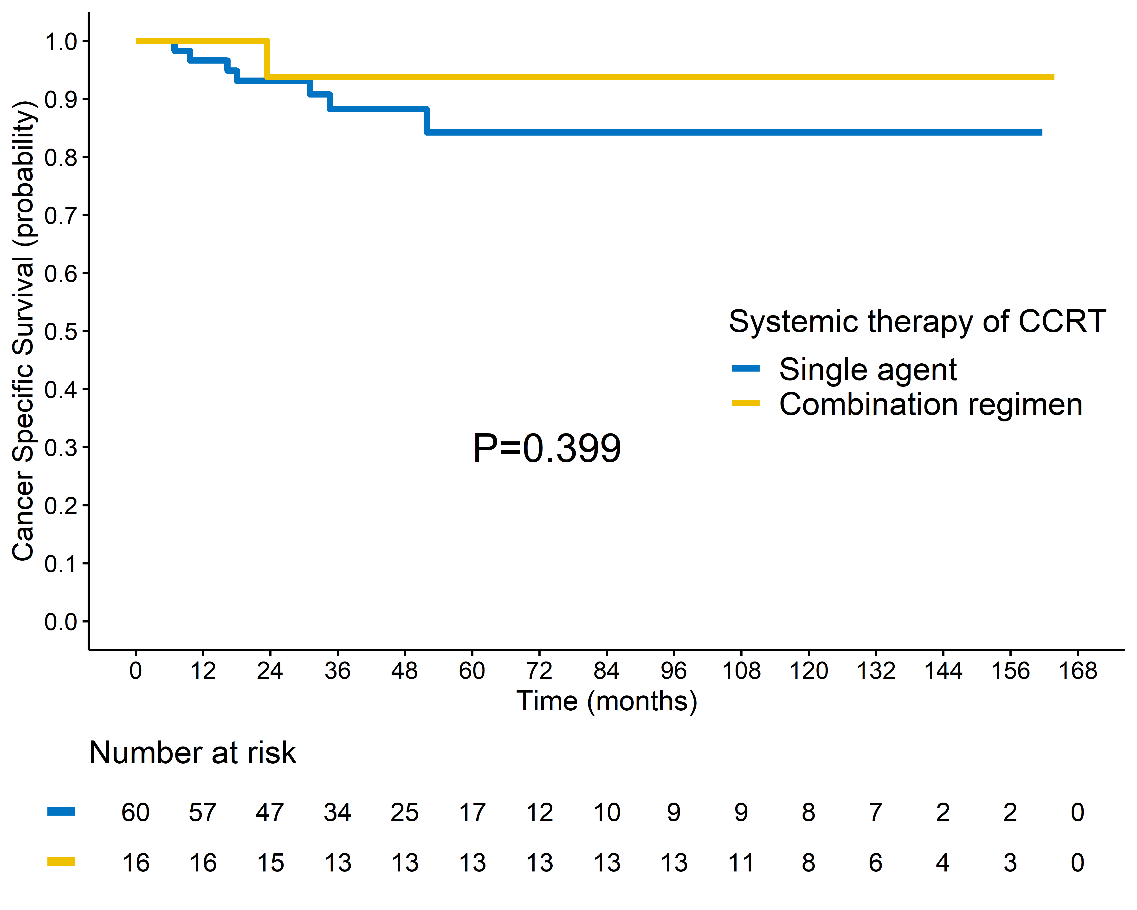


(B)


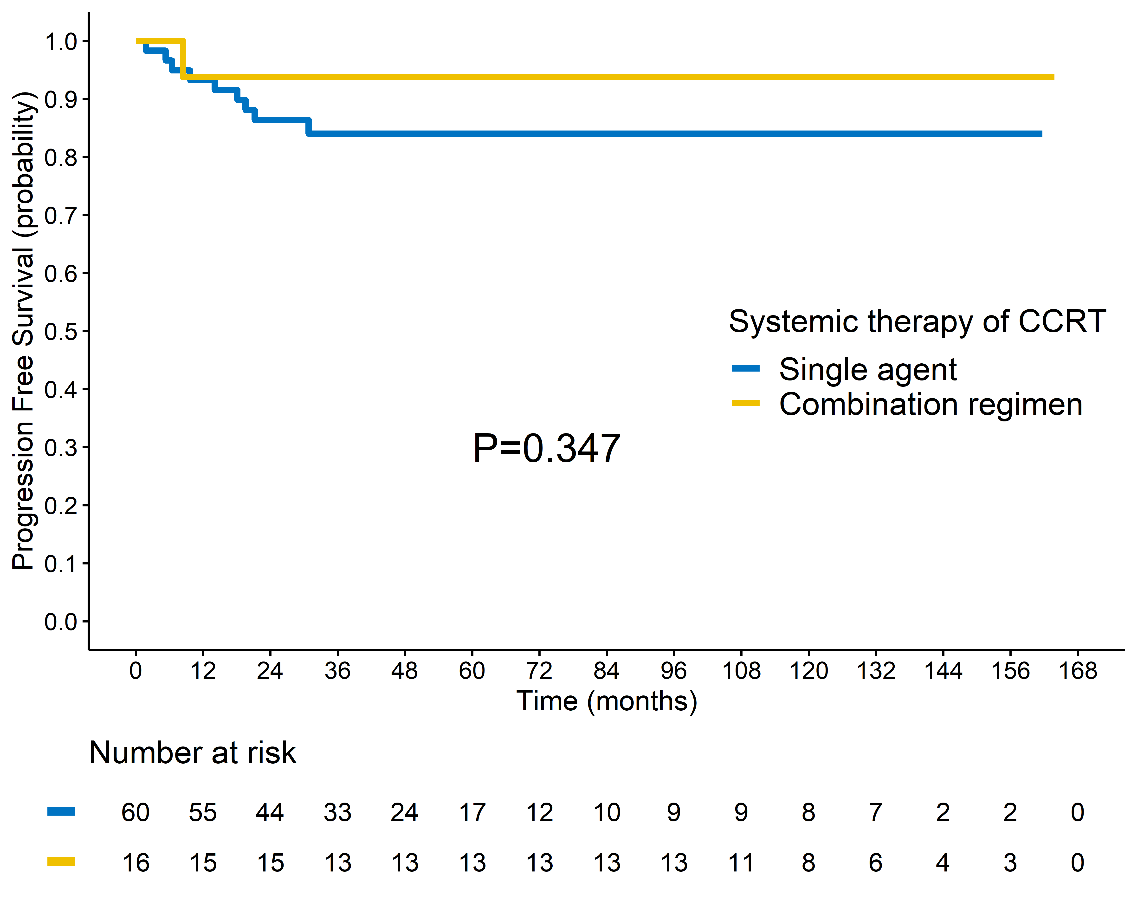


Supplementary Figure 6. Survival curves for patients receiving CCRT with single agent versus with combination therapy in the combined cohort with cumulative risk score 1. (A) Cancer-free survival. (B) Progression-free survival.

(A)


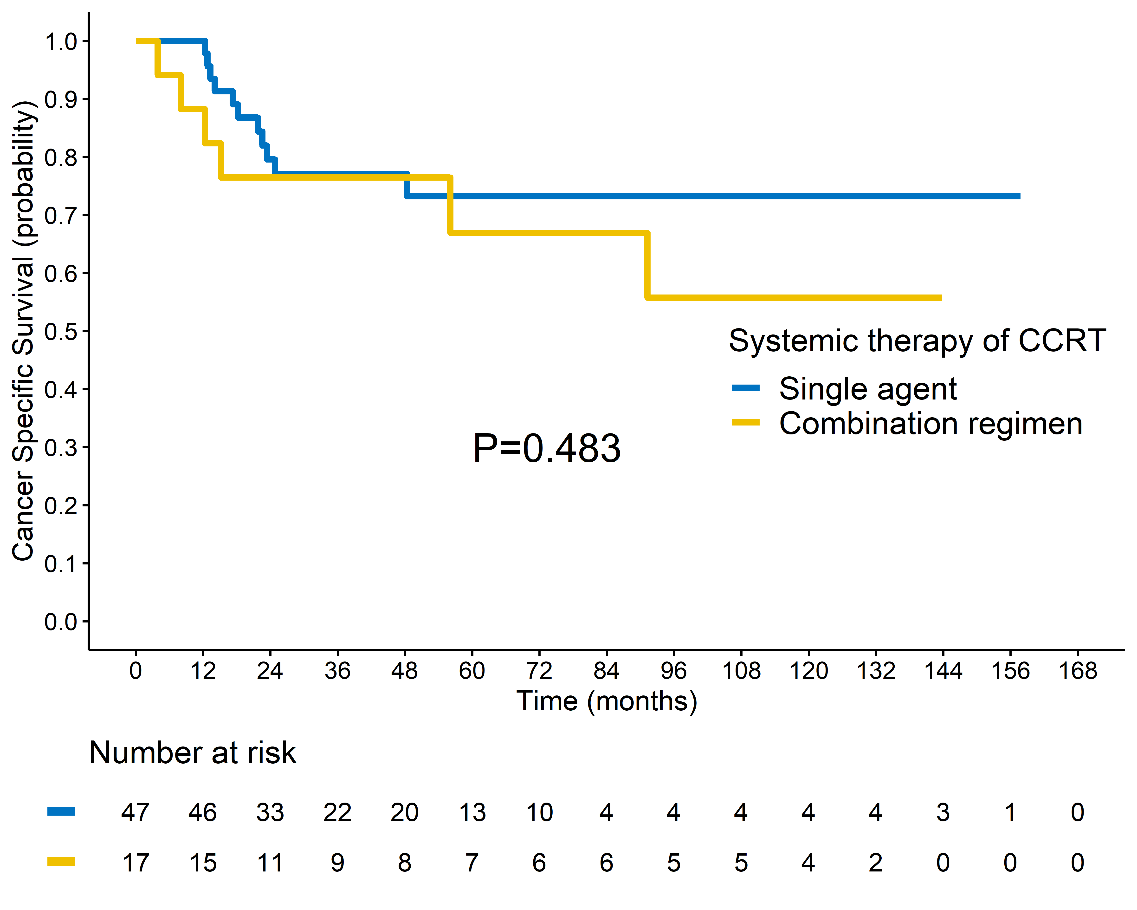


(B)


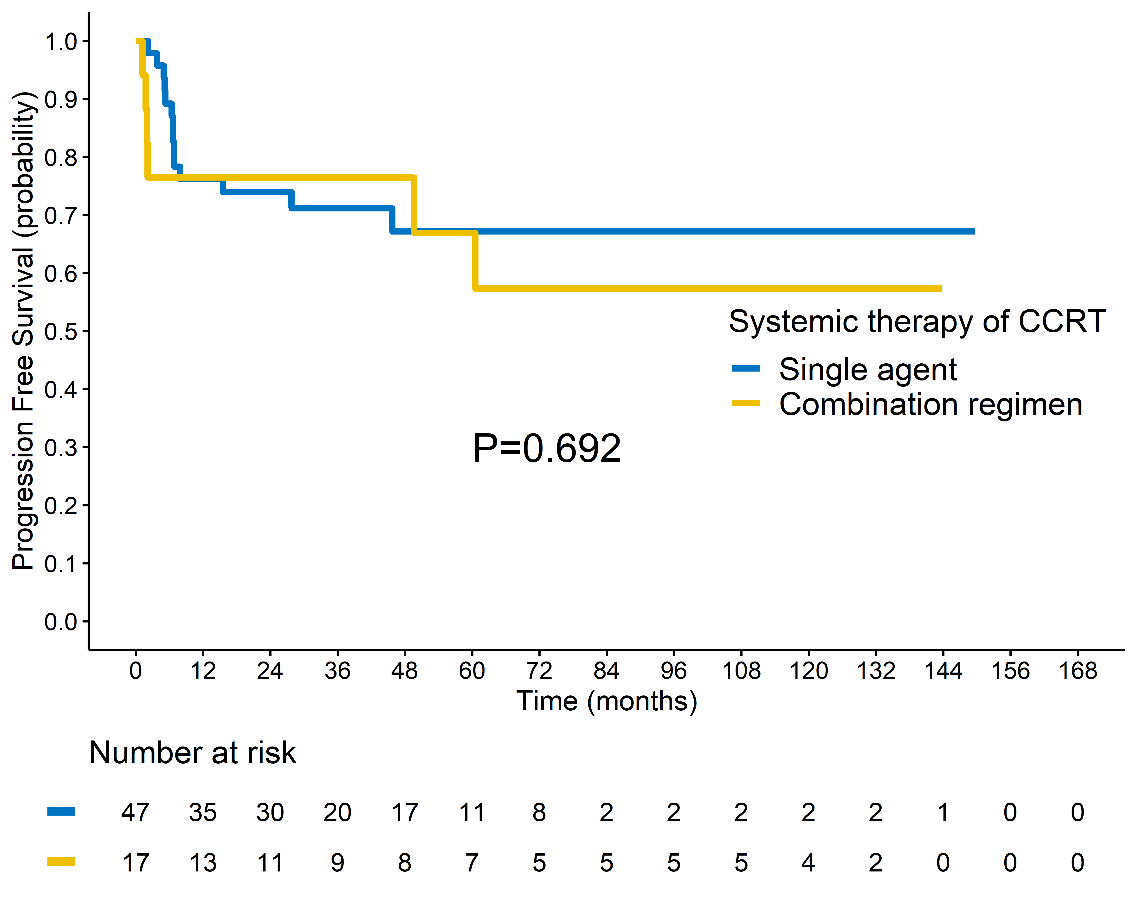


Supplementary Figure 7. Survival curves for patients receiving CCRT with single agent versus with combination therapy in the combined cohort with cumulative risk score 2. (A) Cancer-free survival. (B) Progression-free survival.

(A)


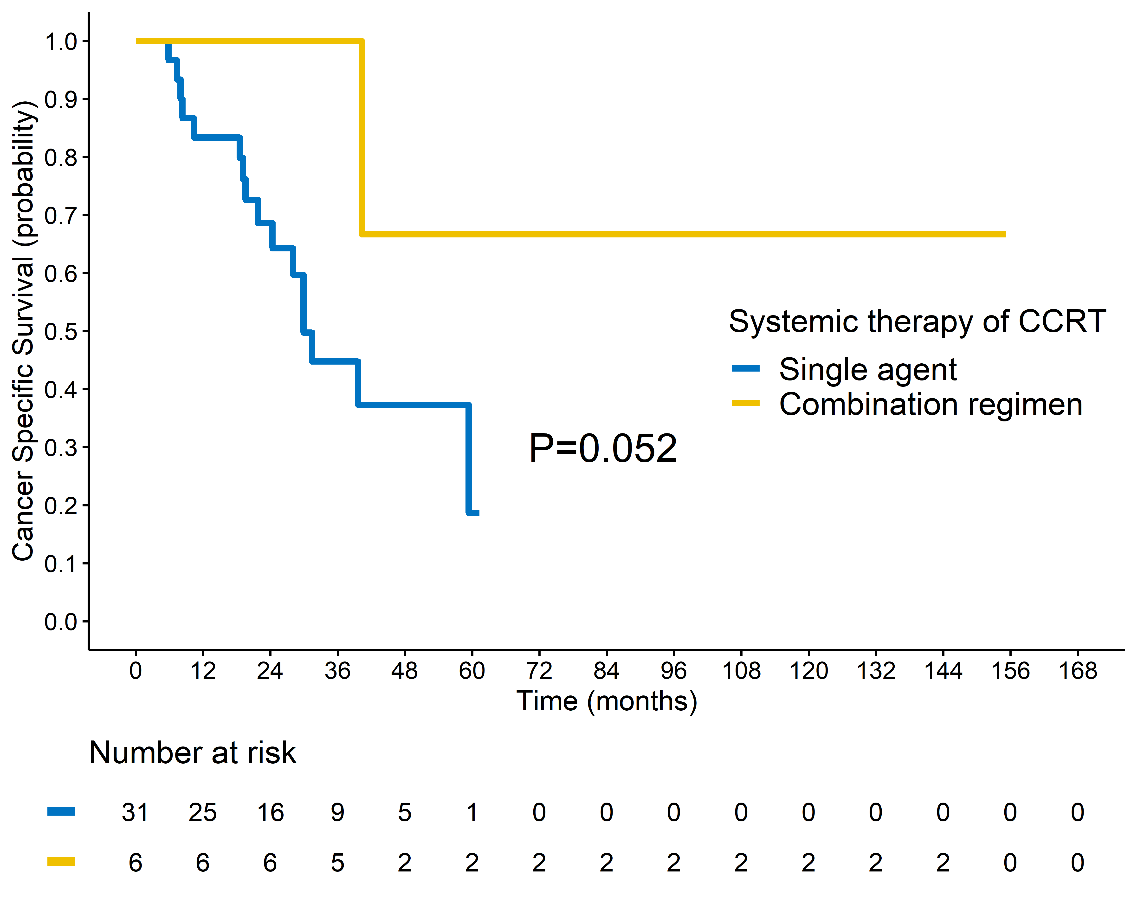


(B)


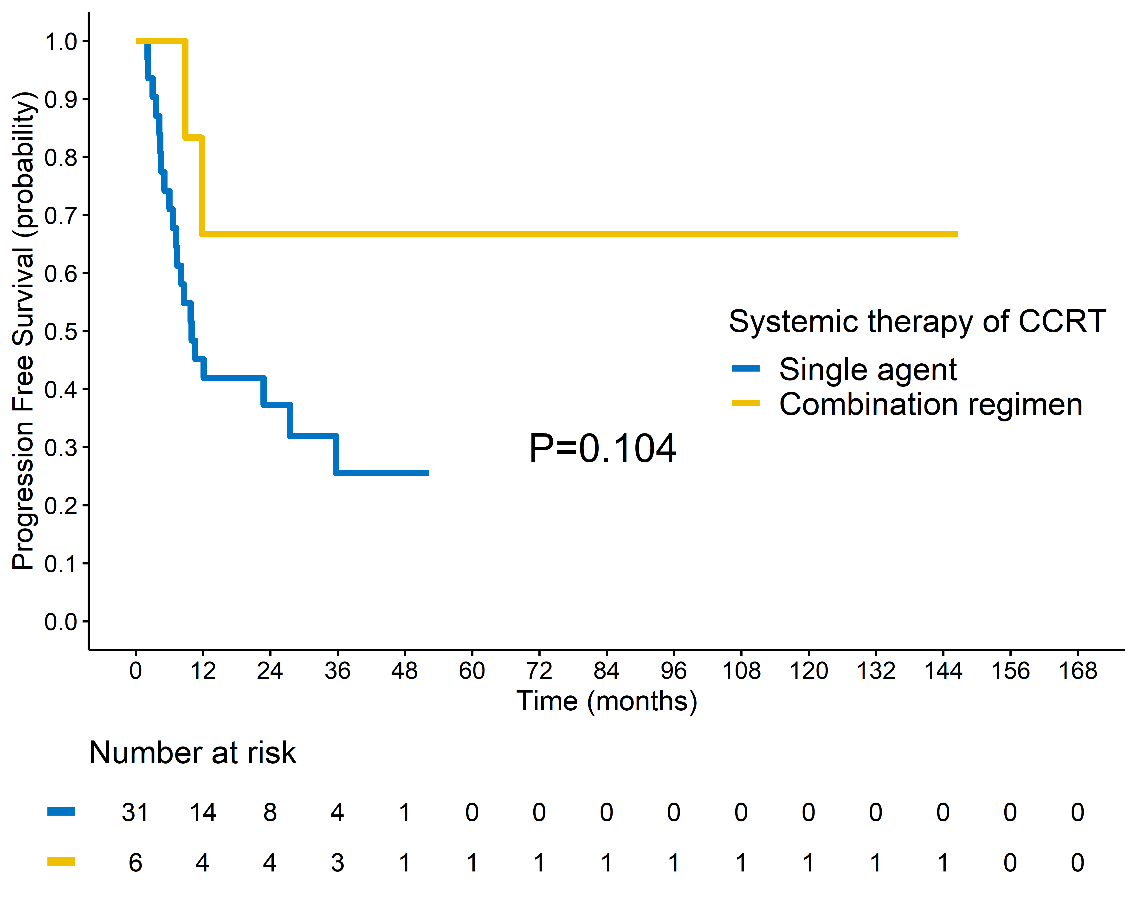


Supplementary Figure 8. Survival curves for patients receiving CCRT with single agent versus with combination therapy in the combined cohort with cumulative risk score 3. (A) Cancer-free survival. (B) Progression-free survival.

(A)


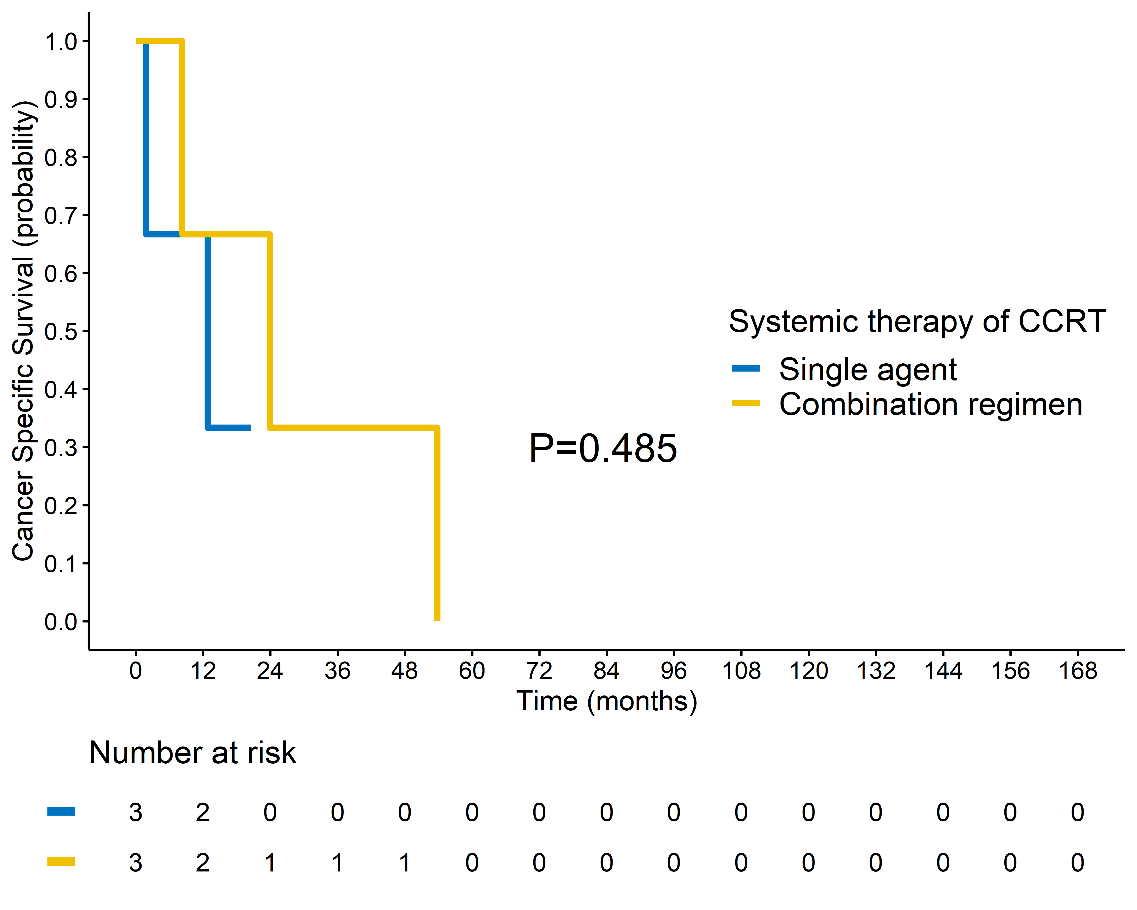


(B)


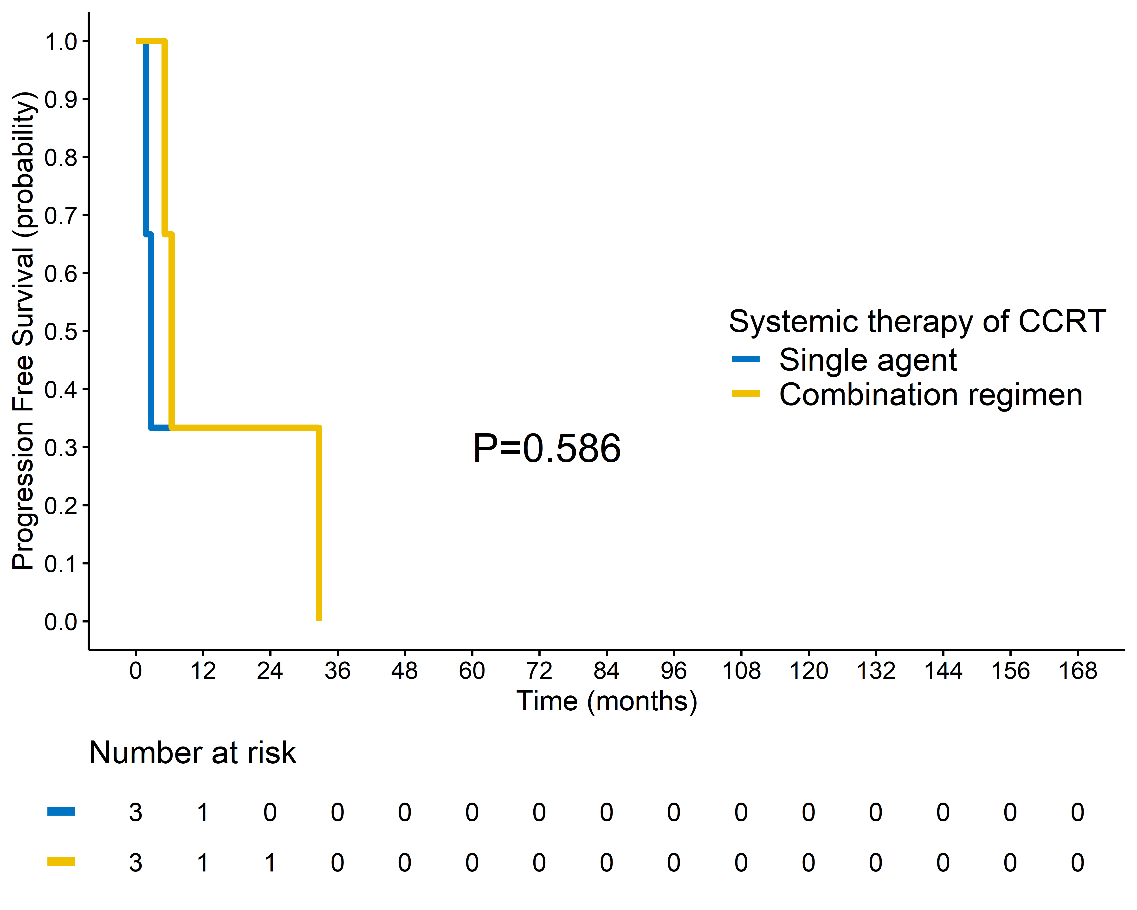

Supplement: Supplementary file 1 — Appendix S1. [file CAM4-13-e6746-s001.docx]
